# Associations of Habitual Sleep Duration and Sleep Stages With Speech-in-Noise Performance

**DOI:** 10.1093/geroni/igab046.131

**Published:** 2021-12-17

**Authors:** Kening Jiang, Adam Spira, Kelsie Full, Emmanuel Garcia, Frank Lin, Nicholas Reed, Pamela Lutsey, Jennifer Deal

**Affiliations:** 1 Johns Hopkins Cochlear Center for Hearing and Public Health, Baltimore, Maryland, United States; 2 Johns Hopkins Bloomberg School of Public Health, Baltimore, Maryland, United States; 3 Division of Epidemiology and Community Health, University of Minnesota, Minneapolis, Minnesota, United States; 4 Johns Hopkins University, Johns Hopkins University, Maryland, United States; 5 School of Public Health, University of Minnesota, Minneapolis, Minnesota, United States; 6 Johns Hopkins University, Baltimore, Maryland, United States

## Abstract

Speech-in-noise performance involves central auditory and cortical processing and is fundamental to communication. We investigated cross-temporal associations of habitual sleep duration and stages (1996-1998) with speech-in-noise performance (2016-2017) in a subset of the Atherosclerosis Risk in Communities Study participated in the Sleep Heart Health Study(N=755, 61±5 years, 53% female). Speech-in-noise performance was measured by Quick Speech-in-Noise Test; range:0-30; lower scores=worse performance. Time spent in each stage (stage 1;2;3/4;rapid eye movement (REM)) was measured by polysomnography. Habitual sleep duration was calculated by self-reported duration on weekdays and weekends. In models adjusting for demographic and disease covariates, every 10-minute increase in REM sleep was associated with better speech-in-noise performance (0.10 points,95% CI:0.00,0.21); every 1-hour increase in habitual sleep duration was associated with worse speech-in-noise performance (-1.28 points,95% CI:-2.49,-0.08) among participants sleep >8 hours. Long sleep duration might be a risk marker of speech-in-noise performance, but REM sleep might be a protective factor.

